# Supplementary material for: Prosocial and antisocial choices in a monogamous cichlid with biparental care
Source: Nat Commun. 2021 Mar 19;12:1775. doi: 10.1038/s41467-021-22075-6 (PMC7979913; doi:10.1038/s41467-021-22075-6)
Supplement: Supplementary file 1 — Supplementary Information [file 41467_2021_22075_MOESM1_ESM.pdf]

Supplemental information

Other-regarding preference in fish: Evidence from a prosocial choice task in a monogamous cichlid

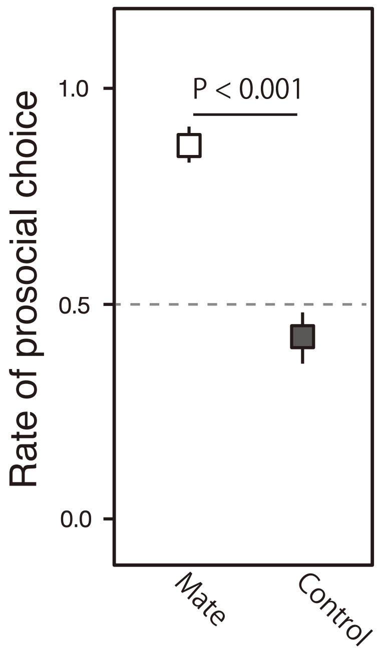

	Estimate ± SD	df	χ^2	P
intercept	− 0.986 ± 0.397	−	−	−
exp	2.753 ± 0.441	1	17.594	< 0.001
order	0.920 ± 0.425	1	3.817	0.051

Supplemental figure S1: Difference in the prosocial choice rate over the last 5 days between mate (female) and control treatment. The analysis was performed with a binomial GLMM, using only data for subjects that had experienced both treatments (mate experiment, n = 6; control experiment n = 6). Broken line at 0.5 represents the null hypothesis of random choice. Data are mean and 95 % CI. Statistical test was two tailed. Statistical information was shown in table.

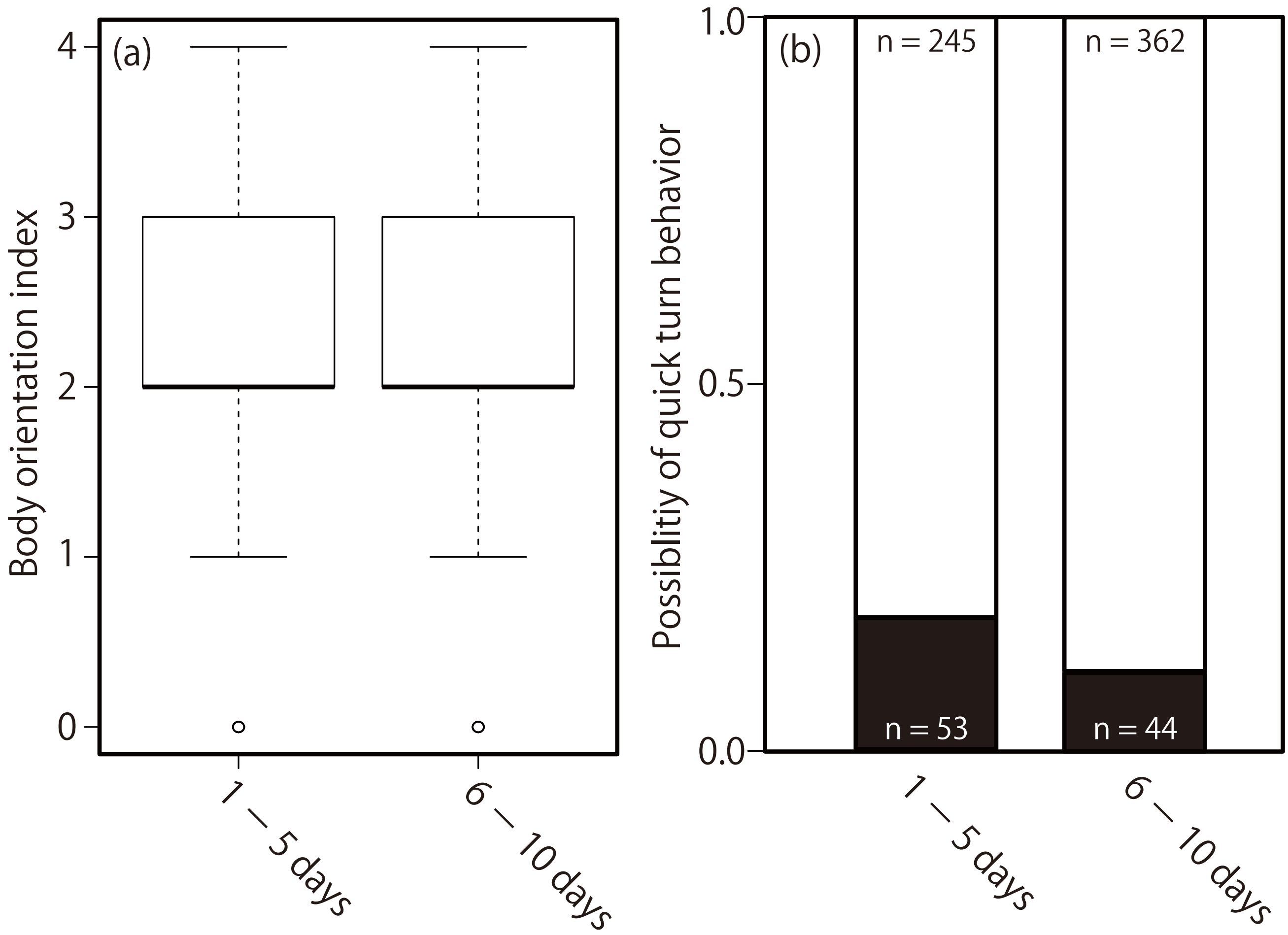

Supplemental figure S2: (a) Differences between 1 the first (*n* = 298 trials from 12 subject males) and the last five days (*n* = 406 trials from 12 subject males) regarding the body orientation index by the subject males when subjects chose the prosocial compartment. Boxplot shows the median and 25th and 75th percentiles; the whiskers extend to the maximum and minimum values and the circles are outliers. (b) The probability of subjects showing quick turn behavior towards their foraging mate during the first (*n* = 298 trials from 12 subject males) and the last five days (*n* = 406 trials from 12 subject males) of experiments. Bars shows mean ± SD.

Supplemental table S3: Factors affecting the probability of a prosocial choice of subject male convict cichlids *Amatitlania nigrofasciata* in the treatment with the mate as recipient. Only experimental day had a significant effect, while the percentage of time spent in front of both compartments (= time spent of prosocial or antisocial compartment / time of the trial) during trial did not affect male choices. Statistical test was two tailed.

**terms**	***df***	χ^2	***P***
days	1	147.945	< 0.001
% of time spent in front of prosocial compartment	1	1.039	0.308
% of time spent in front of antisocial compartment	1	0.705	0.401

Supplemental table S4: Factors affecting the probability of a prosocial choice of subject male convict cichlids *Amatitlania nigrofasciata* in the treatment with the mate as recipient. Only experimental day had a significant effect, while the position of the mate in her tank (far from the male's boxes, in front of the male's prosocial choice or in front of the male's antisocial choice) did not affect male choices. Statistical test was two tailed.

**terms**	***df***	χ^2	***P***
days	1	147.945	< 0.001
position of mate female (in front of prosocial compartment, antisocial compartment, and far from compartmets)	2	1.574	0.455

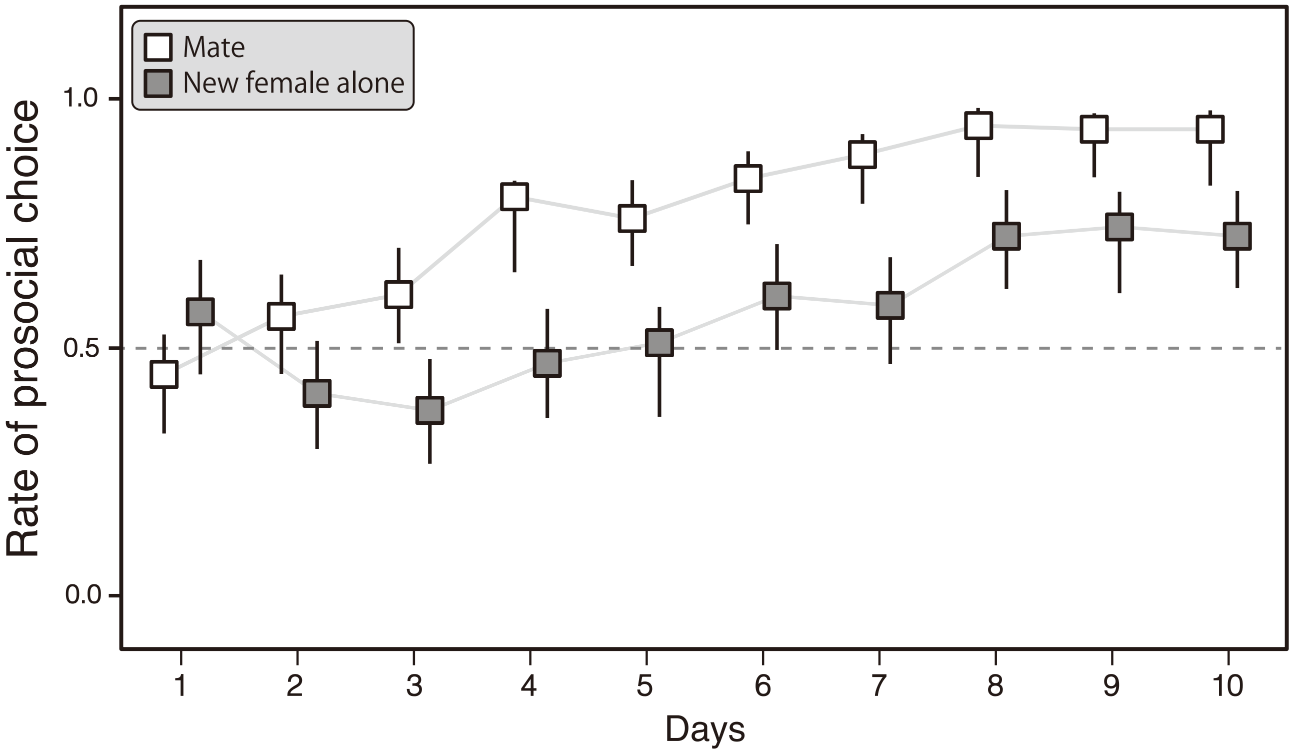

Supplemental figure S5. Change in the prosocial choice rate during the mate experiment (white squares, n = 12) and the new female alone experiment (grey squares, n =10). Broken line at 0.5 represents the null hypothesis of random choice. Data are mean and 95 % CI.

Supplemental table S6: Experimental order of PCT with different treatments.

ID	Experimenter	Order 1	Order 2	Order 3	Order 4	Order 5
I1	S.I.	mate	rival male	control		
I2	S.I.	control				
I3	S.I.	mate	new female[b]	new female[a]	rival male	
I4	S.I.	new female[b]	control			
I5	S.I.	control	mate			
I6	S.I.	new female[a]				
I7	S.I.	control				
I8	S.I.	control				
M1	M.S.	mate				
S1	S.S.	mate	control			
S2	S.S.	mate				
S3	S.S.	mate	control	rival male	new female[b]	new female[a]
S4	S.S.	new female[a]	mate	rival male	new female[b]	
S5	S.S.	new female[a]	rival male	mate	control	
S6	S.S.	control	mate	rival male		
S7	S.S.	mate				
S8	S.S.	mate				
S10	S.S.	new female[a]	control	new female[b]	rival male	
S11	S.S.	new female[b]	rival male	control	new female[a]	
S12	S.S.	rival male	new female[b]	new female[a]		
S13	S.S.	new female[b]	new female[a]			
S14	S.S.	rival male				
S15	S.S.	new female[a]	new female[b]			
S16	S.S.	new female[b]				

[a] new female alone, [b] new female with subject's mate present

Supplementary Movie 1: Typical examples of quick tune behavior by subject during choice experiment.
