# Supplementary material for: Prosocial and antisocial choices in a monogamous cichlid with biparental care
Source: Nat Commun. 2021 Mar 19;12:1775. doi: 10.1038/s41467-021-22075-6 (PMC7979913; doi:10.1038/s41467-021-22075-6)
Supplement: Supplementary file 3 — Reporting Summary [file 41467_2021_22075_MOESM3_ESM.pdf]

nature research

Corresponding author(s): Shun Satoh

Last updated by author(s): Jan 20, 2021

Reporting Summary

Nature Research wishes to improve the reproducibility of the work that we publish. This form provides structure for consistency and transparency in reporting. For further information on Nature Research policies, see our Editorial Policies and the Editorial Policy Checklist.

Statistics

For all statistical analyses, confirm that the following items are present in the figure legend, table legend, main text, or Methods section.

n/a	Confirmed	
☐	☒	The exact sample size (n) for each experimental group/condition, given as a discrete number and unit of measurement
☐	☒	A statement on whether measurements were taken from distinct samples or whether the same sample was measured repeatedly
☐	☒	The statistical test(s) used AND whether they are one- or two-sided<br>*Only common tests should be described solely by name; describe more complex techniques in the Methods section.*
☐	☒	A description of all covariates tested
☐	☒	A description of any assumptions or corrections, such as tests of normality and adjustment for multiple comparisons
☐	☒	A full description of the statistical parameters including central tendency (e.g. means) or other basic estimates (e.g. regression coefficient) AND variation (e.g. standard deviation) or associated estimates of uncertainty (e.g. confidence intervals)
☐	☒	For null hypothesis testing, the test statistic (e.g. F, t, r) with confidence intervals, effect sizes, degrees of freedom and P value noted<br>*Give P values as exact values whenever suitable.*
☒	☐	For Bayesian analysis, information on the choice of priors and Markov chain Monte Carlo settings
☐	☒	For hierarchical and complex designs, identification of the appropriate level for tests and full reporting of outcomes
☒	☐	Estimates of effect sizes (e.g. Cohen's d, Pearson's r), indicating how they were calculated

Our web collection on statistics for biologists contains articles on many of the points above.

Software and code

Policy information about availability of computer code

Data collection	No software was used for the data collection of this study.
Data analysis	R software v. 3.1.1. (44. R Core Team. R: a language and environment for statistical computing. R Foundation for Statistical Computing, Vienna, Austria. https://www.R-project.org/)

For manuscripts utilizing custom algorithms or software that are central to the research but not yet described in published literature, software must be made available to editors and reviewers. We strongly encourage code deposition in a community repository (e.g. GitHub). See the Nature Research guidelines for submitting code & software for further information.

Data

Policy information about availability of data

All manuscripts must include a data availability statement. This statement should provide the following information, where applicable:

- Accession codes, unique identifiers, or web links for publicly available datasets
- A list of figures that have associated raw data
- A description of any restrictions on data availability

The behavioral data that support the findings of this study are available in “dryad” doi:10.5061/dryad.k3j9kd565

Field-specific reporting

Ecological, evolutionary & environmental sciences study design

All studies must disclose on these points even when the disclosure is negative.

Study description	To test whether male convict cichlid Amatitlania nigrofasciata has prosocility, we performed prosocial choice task (PCT) inspired by study of primates. We performed choice experiments with five treatments (control, mate, rival mate, new female alone, and new female with presentation experiments). In total, 54 choice experiments were performed (control n = 12, mate n = 12, rival male n = 10, new female alone n =10, new female with presentation n = 10) to test whether social relationship and situation affect their prosociality. In this experiment, we provided two option, 1) selfish and 2) prosocial choice. If subject chose prosocial choice, subject and presented fish could consume foods. However, if subject chose selfish choice, only subject could consume foods. We compared rate of prosocial choice in each treatment with control (presented fish was absent).
Research sample	Convict cichlid Amatitlania nigrofasciata (size, 8–10 cm), male fish (n = 30 in total) and female fish (n = 36), we bought them by ornamental fish companies. We used this fish because body size is smaller and easy to breed. Age was unknown but they were sexually matured.
Sampling strategy	We used convict cichlid Amatitlania nigrofasciata (size, 8–10 cm), male fish (n = 30 in total) and female fish (n = 36 in total). We performed 54 choice experiments and observed their behavior for 704 trials. Sample sizes were decided before experiment started. Validity of sample sizes were also statistically confirmed in the analysis. We bought them from ornament fish companies. To ensure homogeneity in kin relationship between the subject male and presented fish, different family lines of adult males and females were present in some aquarium.
Data collection	All experiments and behavioral observations were conducted in Osaka City University (OCU), Osaka prefecture, Japan. Three authors (S.S., M.S., S.I.) performed experiments. First of all, we performed choice experiment with five treatment. During mate experiment, we videotaped behaviors of subject and presented fish. After experiments, a third-person observer who was blind to our hypothesis performed behavioral observation for subject and presented fish.
Timing and spatial scale	Choice experiment: Experiments was conducted between November 2015 and February 2018. We had two to four experimental water tank. Therefore, when experiment was finished, we could began to another experiment. Experiments constantly performed during this period by one to three persons.<br><br>Behavioral observation using video data: Behavioral observations under the blind condition was conducted at 2018.
Data exclusions	If a subject chose less than three times in a day, the data for that day were discarded (n = 2 trials in mate experiment, n = 2 trials control experiment). This exclusion criteria were established before experiment starting.
Reproducibility	Because some subjects showed a strong side bias during the first three days choosing one side > 80% of trials), it was discarded from the experiment and no further trials were conducted. Each treatment consisted of 9 trials per day over 10 consecutive days, yielding a maximum of 90 trials per treatment. We are performing similar experiment and gain some data similar with this study. Therefore, we think reproducibility of present study was confirmed. To avoid observer bias, behavioral observation was performed under blind condition.
Randomization	Fish were haphazardly allocated into five experiments, but as can be seen in supplemental table S6, the order of treatments was not completely randomized for two reasons. This is because we initially focused on the comparison between the treatments 'mate' and 'control', and added the other three treatments only during the ongoing experiment. Second, we did not subject all subjects to all treatments for various reasons, meaning that subjects could have been tested in any possible number of treatments (between one and five). However, effects of experimental order was regulated in statistical analysis.
Blinding	The possibility of blinding in the choice experiment was limited. This is because experimenters should provide rewarding foods for presented fish depending choice by subject. Behavioral observation was performed by blind condition by third person who was blind to our hypotheses and experimental concept. We decided to run two statistical analyses, based on the advice of a statistical expert who was also blind to our hypotheses and only concerned with data quality.

Did the study involve field work? ☐ Yes ☒ No

Reporting for specific materials, systems and methods

We require information from authors about some types of materials, experimental systems and methods used in many studies. Here, indicate whether each material, system or method listed is relevant to your study. If you are not sure if a list item applies to your research, read the appropriate section before selecting a response.

Materials & experimental systems

n/a	Involved in the study
☒	☐ Antibodies
☒	☐ Eukaryotic cell lines
☒	☐ Palaeontology and archaeology
☐	☒ Animals and other organisms
☒	☐ Human research participants
☒	☐ Clinical data
☒	☐ Dual use research of concern

Methods

n/a	Involved in the study
☒	☐ ChIP-seq
☒	☐ Flow cytometry
☒	☐ MRI-based neuroimaging

Animals and other organisms

Policy information about studies involving animals; ARRIVE guidelines recommended for reporting animal research

Laboratory animals	Amatitlania nigrofasciata (size, 8–10 cm), male fish (n = 30 in total) and female fish (n = 36), we bought them by ornamental fish companies. Age was unknown but they were sexually matured.
Wild animals	No wild animals was used for our study.
Field-collected samples	No field-collected samples were used for our study.
Ethics oversight	All experimental protocols were approved by the Animal Care and Use Committees at the Osaka City University for Advanced Studies and adhered to the ASAB/ABS guidelines for the treatment of animals in behavioral research.

Note that full information on the approval of the study protocol must also be provided in the manuscript.
