# Supplementary material for: Prosocial and antisocial choices in a monogamous cichlid with biparental care
Source: Nat Commun. 2021 Mar 19;12:1775. doi: 10.1038/s41467-021-22075-6 (PMC7979913; doi:10.1038/s41467-021-22075-6)
Supplement: Supplementary file 4 — Description of Additional Supplementary Files [file 41467_2021_22075_MOESM4_ESM.pdf]

Supplementary Movie 1: Typical examples of quick tune behavior by subject during choice experiment.
